# Supplementary material for: Yeast Rad52 is a homodecamer and possesses BRCA2-like bipartite Rad51 binding modes
Source: Nat Commun. 2023 Oct 5;14:6215. doi: 10.1038/s41467-023-41993-1 (PMC10556141; doi:10.1038/s41467-023-41993-1)
Supplement: Supplementary file 3 — Reporting Summary [file 41467_2023_41993_MOESM3_ESM.pdf]

Corresponding author(s): Edwin Antony

Last updated by author(s): Sep 22, 2023

Reporting Summary

Nature Portfolio wishes to improve the reproducibility of the work that we publish. This form provides structure for consistency and transparency in reporting. For further information on Nature Portfolio policies, see our Editorial Policies and the Editorial Policy Checklist.

Statistics

For all statistical analyses, confirm that the following items are present in the figure legend, table legend, main text, or Methods section.

n/a	Confirmed	
☐	☒	The exact sample size (*n*) for each experimental group/condition, given as a discrete number and unit of measurement
☐	☒	A statement on whether measurements were taken from distinct samples or whether the same sample was measured repeatedly
☒	☐	The statistical test(s) used AND whether they are one- or two-sided<br>*Only common tests should be described solely by name; describe more complex techniques in the Methods section.*
☒	☐	A description of all covariates tested
☒	☐	A description of any assumptions or corrections, such as tests of normality and adjustment for multiple comparisons
☐	☒	A full description of the statistical parameters including central tendency (e.g. means) or other basic estimates (e.g. regression coefficient) AND variation (e.g. standard deviation) or associated estimates of uncertainty (e.g. confidence intervals)
☒	☐	For null hypothesis testing, the test statistic (e.g. *F*, *t*, *r*) with confidence intervals, effect sizes, degrees of freedom and *P* value noted<br>*Give P values as exact values whenever suitable.*
☒	☐	For Bayesian analysis, information on the choice of priors and Markov chain Monte Carlo settings
☒	☐	For hierarchical and complex designs, identification of the appropriate level for tests and full reporting of outcomes
☒	☐	Estimates of effect sizes (e.g. Cohen's *d*, Pearson's *r*), indicating how they were calculated

Our web collection on statistics for biologists contains articles on many of the points above.

Software and code

Policy information about availability of computer code

Data collection

cryoEM:

Structure Determination:

Fluorescence: Steady-state fluorescence data were collected on an PC1 fluorimeter (ISS Inc.) using the instrument-associated Vinci 3 software.
TIRF microscopy

Stopped Flow: Stopped flow kinetics data were collected using a SX-20 instrument (Applied Photophysics Inc.) and instrument-associated software provided by Applied Photophysics (Pro-Data SX ver.2.5.1852.0).

XL-MS and HDX-MS: In Mass Spectrometry based deuterium exchange experiments, peptides were identified using MassHunter Qualitative Analysis, version 6.0 (Agilent Technologies), Peptide Analysis Worksheet (ProteoMetrics LLC), and Pep-tideShaker, version 1.16.42, paired with SearchGUI, version 3.3.16 (CompOmics).

AUC: Analytical ultracentrifugation experiments were collected using the instrument associated software (Beckman Inc) and exported data were fit and analyzed using SEDFIT.

Mass Photometry:
Gel Imaging: Gels were scanned using an iBright-1500 imager (Thermo Fisher Scientific) and acquired using iBright software (ver.3.0.1).
Circular Dichroism: Circular Dichroism data were collected on a Chirascan V100 instrument (Applied Photophysics Inc) using the associated Pro-Data Chirascan software (ver.4.7.0.194).

April 2023

Single-molecule analysis: We have uploaded the code for single molecule analysis in Github. Here is the link: https://github.com/ChaddaRah/Single-Molecule-Trajectory-Analyzer_RahulChadda.https://github.com/ChaddaRah/Single-Molecule-Trajectory-Analyzer_RahulChadda.

Data analysis	Quantitative Date: Numerical data were exported and analyzed and fitted using GraphPad Prism 9 (Ver. 9.4.0 (673)) or Kaleidagraph (Ver. 4.5).<br>Fluorescence: Steady-state fluorescence data were analyzed using GraphPad Prism 9 (Ver. 9.4.0 (673)).<br><br>HDX-MS: The Mass Spectrometry based deuterium exchange experiments, peptides were identified using MassHunter Qualitative Analysis, version 6.0 (Agilent Technologies), Peptide Analysis Worksheet (ProteoMetrics LLC), and Pep-tideShaker, version 1.16.42, paired with SearchGUI, version 3.3.16 (CompOmics).<br>Stopped Flow: Stopped flow data were analyzed using Kaleidagraph (Ver. 4.5).<br><br>cryoEM:<br><br>Structure Determination:<br><br>TIRF microscopy<br><br>XL-MS: Crosslinks were then determined using Spectrum Identification Machine (SIMXL 1.5.5.2).<br><br>AUC:<br><br>Mass Photometry:

For manuscripts utilizing custom algorithms or software that are central to the research but not yet described in published literature, software must be made available to editors and reviewers. We strongly encourage code deposition in a community repository (e.g. GitHub). See the Nature Portfolio guidelines for submitting code & software for further information.

Data

Policy information about availability of data

All manuscripts must include a data availability statement. This statement should provide the following information, where applicable:

- Accession codes, unique identifiers, or web links for publicly available datasets
- A description of any restrictions on data availability
- For clinical datasets or third party data, please ensure that the statement adheres to our policy

The coordinates for the Rad52 structure are available in the PDB with code 8G3G and the Cryo-EM maps are available in EMBD with code EMD-29695. The source data for all experiments shown are provided as a Source Data file. Constructs for protein expression are available from the corresponding author upon request.

Research involving human participants, their data, or biological material

Policy information about studies with human participants or human data. See also policy information about sex, gender (identity/presentation), and sexual orientation and race, ethnicity and racism.

Reporting on sex and gender	N/A
Reporting on race, ethnicity, or other socially relevant groupings	N/A
Population characteristics	N/A
Recruitment	N/A
Ethics oversight	N/A

Note that full information on the approval of the study protocol must also be provided in the manuscript.

Field-specific reporting

Please select the one below that is the best fit for your research. If you are not sure, read the appropriate sections before making your selection.

☒ Life sciences ☐ Behavioural & social sciences ☐ Ecological, evolutionary & environmental sciences

For a reference copy of the document with all sections, see nature.com/documents/nr-reporting-summary-flat.pdf

Life sciences study design

All studies must disclose on these points even when the disclosure is negative.

Sample size	All experiments were repeated a minimum of three times with atleast two independent protein preparations.
Data exclusions	No data collected were excluded from analysis.
Replication	Experiments were repeated n=3 or more independently.
Randomization	N/A
Blinding	N/A

Reporting for specific materials, systems and methods

We require information from authors about some types of materials, experimental systems and methods used in many studies. Here, indicate whether each material, system or method listed is relevant to your study. If you are not sure if a list item applies to your research, read the appropriate section before selecting a response.

Materials & experimental systems

n/a	Involved in the study
☒	☐ Antibodies
☒	☐ Eukaryotic cell lines
☒	☐ Palaeontology and archaeology
☒	☐ Animals and other organisms
☒	☐ Clinical data
☒	☐ Dual use research of concern
☒	☐ Plants

Methods

n/a	Involved in the study
☒	☐ ChIP-seq
☒	☐ Flow cytometry
☒	☐ MRI-based neuroimaging
