# Supplementary material for: Yeast Rad52 is a homodecamer and possesses BRCA2-like bipartite Rad51 binding modes
Source: Nat Commun. 2023 Oct 5;14:6215. doi: 10.1038/s41467-023-41993-1 (PMC10556141; doi:10.1038/s41467-023-41993-1)
Supplement: Supplementary file 4 — Source Data [file 41467_2023_41993_MOESM4_ESM.zip › Source_Data/Inventory_of_Source_Data.docx]

**Inventory of Source Data file**

Source Data for main figures

Source Data Supplementary Information

Chimera session file for Rad52-Rad51 complex analysis
